# A de-identified database of 11,979 verbal autopsy open-ended responses

**DOI:** 10.12688/gatesopenres.12812.1

**Published:** 2018-04-17

**Authors:** Abraham D. Flaxman, Lisa Harman, Jonathan Joseph, Jonathan Brown, Christopher J.L. Murray

**Affiliations:** 1 Institute for Health Metrics and Evaluation, University of Washington, Seattle, WA, 98121, USA

**Keywords:** Verbal autopsy, cause of death, natural language processing, open data

## Abstract

As part of the Gates Grand Challenge 13, the Population Health Metrics Research Consortium (PHMRC) collected data to enable the development and validation of methods that measure cause-specific mortality in populations with incomplete or inadequate cause of death coding.

This work yielded 11,979 verbal autopsy interviews (VAIs). In each, a field interviewer spoke with an individual familiar with the deceased and their final illness, and used a semi-structured questionnaire to collect information about the symptoms of the deceased in their final illness. The VAI collected demographic characteristics, possible risk factors (such as tobacco use), and other potentially contributing characteristics. It also included the open-ended question, *“Could you please summarize, or tell us in your own words, any additional information about the illness and/or death of your loved one?”* (open narrative).

The VAI data were released in a de-identified format in September 2013 through the Global Health Data Exchange, in files that contain verbal autopsies that were collected at six sites in four countries (India, Mexico, Tanzania, and the Philippines).

Due to research interest, we have now created redacted versions of the open narratives from the open-ended question of the questionnaire. We hope that this database will be the source of innovations that increase our knowledge about the causes of ill health and, through this knowledge, produce improvements in health for individuals and populations.

## Introduction

Population health information that is both accurate and comprehensive can aid program implementation, monitoring, and evaluation, resource allocation and planning. However, there are currently large gaps in the technologies and measurement methods that are available to generate this information, and this makes it difficult to address health inequities through effective policy ^1^.

The Population Health Metrics Research Consortium (PHMRC) conducted data collection to enable the development and validation of methods that measure cause-specific mortality in populations with incomplete or inadequate cause of death coding. This work produced around 12,000 verbal autopsy interviews (VAIs), in which a relative or someone familiar with the final illness of the deceased, provides information about the signs symptoms of the final illness, as well as demographic characteristics, and information on risk factor exposures (such as tobacco use), and other potentially relevant characteristics ^2^.

The VAI data were released in a de-identified format in September 2013, through the Global Health Data Exchange, in files that contain verbal autopsies from six sites in four countries (India, Mexico, Tanzania, and the Philippines) using a standardized VA questionnaire developed by the PHMRC. The data is organized into three parts corresponding to the questionnaire modules for each age group: neonate, child, and adult. Each VAI in the database is matched with a “gold standard” diagnoses of underlying causes of death, typically identified from medical records, and using stringent diagnostic criteria (such as laboratory, pathology, or medical imaging findings.) ^3^

One portion of a VAI is the “open narrative,” where the respondent has the opportunity to tell, in their own words, what happened during the illness that led to the death being investigated. This was collected as a final question in the PHMRC survey, after the structured interview, when the respondent was asked, *“Could you please summarize, or tell us in your own words, any additional information about the illness and/or death of your loved one?”* The full response to this question was transcribed and translated into English, and the 2013 data release included counts of stemmed keywords as variables in the final dataset, to allow researchers access to this rich source of unstructured data, while also removing any potentially personally identifiable information (PII) in that portion of the interview.

Due to research interest, we have now created redacted versions of 11,979 open narratives to allow researchers the opportunity to learn even more about how deaths are described. We hope that this database will be the source of innovations that increase our knowledge about the causes of ill health and through knowledge produce improvements in health for individuals and populations.

## Methods

The process of collecting the VAIs has been described in detail previously ^1^. In this article, we provide a detailed account of the protocol used to redact personal information from the open-ended question, and therefore allow the release of the full text of the open narrative collected in the VAIs.

Study participants provided their consent to participate with the knowledge that “reports of the data … will not identify any individual person.” We chose also to redact the names of specific health facilities to avoid the risk of identifying individual health service providers indirectly, through their association with individual facilities. To retain the most information possible for future research, we replaced PII with “tags” that denote what sort of information has been redacted.

An example makes this clear: a typical text was redacted to read, “vaginal bleeding and delay to receive care at [HOSPITAL] was the main cause of death. he said that his wife arrive at the hospital at 8pm and didn’t receive any care until 8am.” Instead of including the name of the specific hospital, we redacted it to [HOSPITAL]. The tags used to replace PII are [HOSPITAL], [DOCTOR], [PATIENT], [PLACE], [PERSON], and [YEAR].

We initially planned to redact dates entirely but chose to redact only the year, to make it easier for future researchers to measure the time between events. To allow for different years, we used the tag [YEAR + n]. An example is “last november of [YEAR]-the deceased got stroke left side of his body. was hospitalized due to high blood pressure last year. january this year was his last hospitalization that leads to death. jan. 26, [YEAR+1]. experienced fast breathing, that’s why he was brought to the hospital (provincial hospital). with oxygen and ngt; got fever and cough; in coma. jan. 31, [YEAR+1]. was tried to revive around 11:00 pm to 12 midnight, but was not able to revive him. around 3:00 am (at dawn), he died.”

When a response referred to multiple different specific hospitals, we redacted the hospitals to [HOSPITAL] and [HOSPITAL2]. Subsequent distinct hospitals in same passage were redacted to [HOSPITAL3], [HOSPITAL4], etc.

We included all VAIs for which there was an open-response string available to redact, even when the response was devoid of information.

We implemented the redaction process in a spreadsheet using Excel 2010, redacted manually by a single data analyst (LH), who read each open-response and replaced each piece of PII with the appropriate tag.

### Redaction rules, with some examples and counterexamples

1. Specific patient becomes [PATIENT]
  Example: John Smith was taken to … -> [PATIENT] was taken to
  Counterexamples (no redaction for the following): She was taken to -> She was taken to (no change)
  My uncle was taken to -> My uncle was taken to (no change)
  The patient was taken to -> The patient was taken to (no change)
2. Specific health facility becomes [HOSPITAL],
3. Specific doctor becomes [DOCTOR]
4. Specific place that is not a health facility becomes [PLACE]
5. Specific person that is not doctor or patient becomes [PERSON]

### Iterative development of redaction rules

We originally planned to redact date (including day, month, and year) to [DATE], but to maintain time sequence, we changed this to not redact entire date where month and/or day show time progression. Only redact [YEAR] to keep the reference to time elapsed. See example below where specific dates show progression of time.

### Examples of [YEAR] redactions

jan. 12, [YEAR]. she was bumped by a motorcycle which seened like it had no lights. the deceased had a little drink at that time and her sense of hearing was poor. she was going to cross the street when that happened. she was brought to the hospital but she was unconscious. her breathing was controlled by a pump. the accident happened at around 6 pm jan. 13, [YEAR]. at around 6 am we found out she's dead because the cardiac monitor showed a straight line.

If progression of time spans over years, [YEAR+n] is used. See example below, where passage refers to following year:

last november of [YEAR]-the deceased got stroke left side of his body. was hospitalized due to high blood pressure last year. january this year was his last hospitalization that leads to death. jan. 26, [YEAR+1]. experienced fast breathing, that’s why he was brought to the hospital (provincial hospital). with oxygen and ngt; got fever and cough; in coma. jan. 31, [YEAR+1]. was tried to revive around 11:00 pm to 12 midnight, but was not able to revive him. around 3:00 am (at dawn), he died.

Where a passage refers to two different hospitals, hospitals are redacted to [HOSPITAL] and [HOSPITAL2]. Subsequent hospitals in same passage would be [HOSPITAL3, [HOSPITAL4], etc:

may 16, [YEAR]. he got accident. was brought immediately to [HOSPITAL] then referred directly to [HOSPITAL2], there his wound was stitched. his head was the affected part. was referred to [HOSPITAL3]. was ct scanned in [HOSPITAL4], there was a break on his forehead. was operated after 2 days. after operation he got fever. the deceased also had cough. as per respondent, it was not just the accident alone who led the deceased to death. there was also a complication of his kidney disease. long before (respondent was not able to remember the exact date), the deceased experienced inability to walk but it was not consulted to the doctor for the deceased doesn't want to. they only went to a traditional healer for treatment. the deceased can't walk for about 7 months but then later on he was able to walk again. after he was also hospitalized at [HOSPITAL5], it was known that he have kidney disease.

### Additional clarifications

Midwife names were redacted to [DOCTOR].

### Dataset validation

We reviewed progress weekly and discussed emerging challenges as they arose. For example, we determined that the original plan of redacting dates entirely to [DATE] seemed to be obscuring valuable information about the time between symptoms. One week later, we determined that our first attempt at a remedy, to include [DATE+days] was to labor intensive, and would prevent redaction from completing within our budget. Our next remedy worked, and that is how we developed the [YEAR+n] approach described above. When redaction was completed, we reviewed a simple random sample of redacted texts and confirmed that all were devoid of PII.

## Ethics approval

This study was approved by the Human Subjects Division of the University of Washington (application number 34413). Ethical approval sought for the VAIs is stated in 1. All data were collected with informed verbal consent from participants before beginning the interview.

## Data availability

Data underlying the study are available on OSF: http://doi.org/10.17605/OSF.IO/XUK5Q ^4^

Data are available under the terms of the Creative Commons Zero "No rights reserved" data waiver (CC0 1.0 Public domain dedication).
