# Supplementary material for: A de-identified database of 11,979 verbal autopsy open-ended responses
Source: Gates Open Res. 2018 Apr 17;2:18. [Version 1] doi: 10.12688/gatesopenres.12812.1 (PMC6030395; doi:10.12688/gatesopenres.12812.1)
Supplement: Peer review history

# Peer review: Referee response for version 1

**DOI:** 10.21956/gatesopenres.13879.r26471

**Published:** 2018-06-14

**Authors:** John Hart

**Affiliations:** 1 Department of Clinical Research, London School of Hygiene and Tropical Medicine, London, UK; 2 Melbourne School of Population and Global Health, The University of Melbourne, Melbourne, Vic, Australia

The authors present the release of narrative free text from verbal autopsy (VA) interview data collected as part of the PHMRC gold standard VA validation study. The methods describe the rules and processes for redacting personally identifiable information.

 The release of the data is welcome as including the open narrative in automated VA analysis programs can significantly alter output diagnoses. Better understanding of the pros and cons of using free text, and the appropriate weighting for different sources of free text, is likely to be of further interest to researchers as their methods and technology develop. To illustrate, simple text mining for key words such as "malaria" could correctly provide positive evidence towards a diagnosis if the free text read "She suffered from malaria..." but incorrectly if the free text read "Her malaria tests at the health centre were negative".

 Research may use the narrative free text alone but its use in VA analysis algorithms is likely to complement answers to the structured interview component of VA. This dataset includes the gold standard diagnosis and narrative free text but no link to the structured answers. The authors may wish to comment on this.

 The methods are well described, with appropriate examples, and the released dataset clear to understand whilst minimising risk of individual identification.

 Typos

 Page 3: "Each VAI in the database is matched with a “gold standard” diagnoses of underlying causes of death" rather "Each VAI in the database is matched with a “gold standard” diagnosis of underlying cause of death".

I have read this submission. I believe that I have an appropriate level of expertise to confirm that it is of an acceptable scientific standard.

# Peer review: Referee response for version 1

**DOI:** 10.21956/gatesopenres.13879.r26486

**Published:** 2018-06-07

**Authors:** Michel Garenne

**Affiliations:** 1 MRC/Wits Rural Public Health and Health Transitions Research Unit, School of Public Health, Faculty of Health Sciences, University of the Witwatersrand, Johannesburg, South Africa

This note presents a new database of Verbal Autopsy (VA) narratives gathered by the Population Health Metrics project. The database is placed in open access and is easily accessible; it ensures the confidentiality of the persons who answered the questionnaire.

 This database has a rich potential for further research. Firstly, it can be used for analyzing what caught people’s eye: signs, symptoms and circumstances that people tend report in specific circumstances; for analyzing what was ignored or remained unreported although most likely present according to the medical diagnosis; and for determining who knows about the precise cause of death learned from the medical authorities. This information could be used for better understanding VA narratives. Secondly, it could be used for further refining the VA diagnoses, although this was already done in parts by detecting key words in earlier studies. Thirdly, the database can be used for a full scale textual analysis, including style of reporting, keywords, underlying emotions, selectivity, etc. Lastly, relating the full scale textual analysis with the medical diagnosis and with the answers to the VA structured questionnaire could be most useful for further research.

 This database is therefore most welcome, and likely to become a source of numerous research exercises.

            

Typos:

1. Page 4: “ *she was bumped by a motorcycle which seened like it had no lights”. *Rather “seemed”
2. In the data file, the codebook for Study Site is duplicated.
3. There are many typos in the narratives (example: diabtes for diabetes, etc.). Could this be cleaned? This would help for searching key words and performing textual analysis. 

I have read this submission. I believe that I have an appropriate level of expertise to confirm that it is of an acceptable scientific standard.
